# A Single *Aspergillus fumigatus* Gene Enables Ergothioneine Biosynthesis and Secretion by *Saccharomyces cerevisiae*

**DOI:** 10.3390/ijms231810832

**Published:** 2022-09-16

**Authors:** Sean Doyle, Daragh D. Cuskelly, Niall Conlon, David A. Fitzpatrick, Ciara B. Gilmartin, Sophia H. Dix, Gary W. Jones

**Affiliations:** 1 Department of Biology, Maynooth University, Maynooth, Co., W23 F2K8 Kildare, Ireland; 2 Kerry Group, Naas, Co., W91 W923 Kildare, Ireland; 3 Pfizer, Grangecastle, Co., D22 V8F8 Dublin, Ireland; 4 Centre for Biomedical Science Research, School of Health, Leeds Beckett University, Leeds LS1 3HE, UK

**Keywords:** antioxidant, AlphaFold, cell factory, redox stress, ROS, ergothioneine

## Abstract

The naturally occurring sulphur-containing histidine derivative, ergothioneine (EGT), exhibits potent antioxidant properties and has been proposed to confer human health benefits. Although it is only produced by select fungi and prokaryotes, likely to protect against environmental stress, the GRAS organism *Saccharomyces cerevisiae* does not produce EGT naturally. Herein, it is demonstrated that the recombinant expression of a single gene, *Aspergillus fumigatus egtA*, in *S. cerevisiae* results in EgtA protein presence which unexpectedly confers complete EGT biosynthetic capacity. Both High Performance Liquid Chromatography (HPLC) and LC–mass spectrometry (MS) analysis were deployed to detect and confirm EGT production in *S. cerevisiae*. The localisation and quantification of the resultant EGT revealed a significantly (*p* < 0.0001) larger quantity of EGT was extracellularly present in culture supernatants than intracellularly accumulated in 96 h yeast cultures. Methionine addition to cultures improved EGT production. The additional expression of two candidate cysteine desulfurases from *A. fumigatus* was thought to be required to complete EGT biosynthesis, namely AFUA_2G13295 and AFUA_3G14240, termed *egt2a* and *egt2b* in this study. However, the co-expression of *egtA* and *egt2a* in *S. cerevisiae* resulted in a significant decrease in the observed EGT levels (*p* < 0.05). The AlphaFold prediction of *A. fumigatus* EgtA 3-Dimensional structure illuminates the bidomain structure of the enzyme and the opposing locations of both active sites. Overall, we clearly show that recombinant *S. cerevisiae* can biosynthesise and secrete EGT in an EgtA-dependent manner which presents a facile means of producing EGT for biotechnological and biomedical use.

## 1. Introduction

The study of ergothioneine (EGT; 2-mercaptohistidine trimethylbetaine) biosynthesis and functionality has attracted extensive interest since the seminal discovery of the EGT biosynthetic genes in *Mycobacterium smegmatis* [1]. It is now clear that the potent antioxidant, which is effectively a sulphurised and tri-aminomethylated derivative of L-His, plays a significant role in protecting the microbial species which produce it against high levels of oxidative stress [2,3]. Moreover, emerging evidence suggests that EGT contributes to redox homeostasis in, and the virulence of, various microorganisms including *Aspergillus fumigatus* and *Mycobacterium tuberculosis* [4,5,6,7,8]. This is in part due to the ability of EGT to attenuate redox stress consequent to its tautomeric thiol and thione forms, with the latter form predominant at physiological pH [9]. In fungi, EGT production appears to be activated by peroxide-induced oxidative stress, especially in *A. fumigatus* and *Neurospora crassa,* and to protect conidia prior to germination, in the latter species [2,3].

EGT is also acquired by animals, including humans, as a dietary component and has been proposed to be a vitamin-like substance [10] which is acquired by cells, and bioaccumulated, through the SLC22A4/OCTN-1 receptor [11]. The antioxidative effects and potential cardioprotective role of EGT, as well as its role in protection against various conditions, have been extensively studied and reviewed [12]. However, one of the most interesting discoveries regarding EGT functionality is in preeclampsia. Specifically, erythrocyte EGT levels in women with preeclampsia were significantly higher than in normotensive pregnant women which suggested its involvement in this critical and devastating condition [13]. Subsequently, the potentially therapeutic effect of EGT in preeclampsia was extensively reviewed in [14], who elegantly argued that the antioxidative effects of EGT could play a role in treating preeclampsia, which comprises oxidative stress components possibly because of mitochondrial dysfunctionality. Subsequently, the therapeutic role of EGT has been studied in a reduced uterine perfusion pressure (RUPP) rat model of preeclampsia, and it was found that hypertension was attenuated, a significantly decreased level of antiangiogenic fms-like tyrosine kinase-1 (sFlt-1) was observed, and mitochondria-specific H_2_O_2_ levels were significantly reduced in tissue extracts from treated animals [15]. Moreover, in the RUPP model system, it has also been reported that EGT facilitates intact mitochondrial functionality and also reduces preeclampsia-associated inflammatory responses [16]. Fortuitously, the emerging role of this important antioxidant in animals is complemented by advances in microbial EGT biosynthesis and bioproduction potential.

In bacteria, EGT biosynthesis is encoded by five genes (*egtABCDE*) [1]. Firstly, L-His is converted to hercynine via the action of EgtD, in a reaction requiring S-adenosylmethionine (SAM; 3 mol) for L-His tri-aminomethylation. EgtA catalyses γ-glutamylcysteine formation, and in a subsequent γ-glutamylcysteine-dependent reaction involving C–S bond formation, EgtB catalyses hercynine conversion to hercynyl-γ-glutamylcysteine sulfoxide. Aminohydrolase EgtC then removes L-Glu from the γ-glutamylcysteine moiety of hercynyl-γ-glutamylcysteine sulphoxide which leads to hercynylcysteine sulphoxide formation. Finally, the C–S lyase, EgtE, a pyridoxal 5′-phosphate-dependent enzyme, enables EGT formation, along with ammonia and pyruvate side-product production [1]. In fungi, it has been reported that two genes, namely *egt-1* and *egt-2* (a functional ortholog of *egtE*) are required for EGT biosynthesis, whereby Egt-1 catalyses hercynylcysteine sulphoxide formation, followed by the Egt-2-mediated generation of EGT [3,17,18]. Moreover, it is thought that fungal EGT biosynthesis requires L-Cys as a thiol source [19]. It should also be noted that the *A. fumigatus* ortholog of *egt-1*, for which limited structural information is available, is termed *egtA* in accordance with the gene nomenclature convention in this species [8] and comprises at least a methyltransferase and a sulphoxide synthase domain, in addition to a putative sulphatase-modifying factor domain [2]. Interestingly, the production of EGT under anaerobic conditions in the bacterium *Chlorobium limicola* has also been reported [20]. Here, trimethylated-L-histidine is converted to EGT, in the absence of oxygen, by a novel enzyme EanB, and the crystal structure of this novel enzyme has been reported [21]. In addition to EanB, a number of enzymes involved in EGT biosynthesis have been crystallised and their structures solved [22], with the histidine methyltransferase EgtD being the most highly conserved protein across EGT biosynthetic pathways (extensively and expertly reviewed in [23]).

From a biotechnological perspective, EGT is produced chemically or extracted from basidiomycetes and is sold as a food supplement or used as an additive for cosmetic products to enhance effectiveness [24]. It has also been suggested that EGT addition to food products (e.g., meat and fish) could prevent oxidative spoilage, enhance both product appearance and shelf-life, and improve texture and potential health benefits [25,26]. Thus, consequent to the identification of genes encoding EGT biosynthesis, the microbial cell factory production of EGT has been investigated as an alternative to chemical synthetic approaches [27,28,29,30,31,32,33,34] (Supplementary Table S1). Secreted EGT production (657 mg/L scale) has been optimally achieved by engineering three bacterial genes (*M. smegmatis egtDE* and *Methylobacterium brachiatum egtB* in an *Escherichia coli* Δ*metJ* strain, concomitant with L-Cys overproduction [32]. Prior to this, up to five *M. smegmatis* genes (*egtABCDE*) had been required for optimal and secreted EGT biosynthesis (up to 1.3 g/L in a fed-batch bioreactor) in *E. coli*, which had also involved either L-His supplementation (5 mM) or thiosulphate addition [28,30]. EGT bioproduction in *Methylobacterium acquaticum* (at a scale equivalent to 20 mg/L) has also been achieved by increased endogenous *egtBD* copy number (2x), although no product secretion was observed. The engineering of *Aspergillus oryzae* with *egt-1* and *egt-2* from *Neurospora crassa* boosted endogenous EGT production levels (11.5 mg/kg media) to 231 mg/kg media, although transgene expression was not determined, and it is unclear if the EGT produced was secreted or present in mycelia [31]. The engineering of *N. crassa egt-1* and *Claviceps purpurea egt-2* into *Saccharomyces cerevisiae* resulted in 600 mg/L EGT production, up to 30% of which was secreted, using a fed-batch fermentation system [29]. Notably, in this latter study, *egt-1* or -*2* gene expression was not determined, and the co-expression of putative EGT transporters or targeted systems engineering did not significantly improve EGT secretion or production. Thus, although EGT production has been achieved in several recombinant systems, all have involved relatively complex host manipulation or multiple transgene insertions. Here, we show that the introduction of a single gene of fungal origin, *A. fumigatus egtA*, enables complete EGT biosynthesis in, and secretion from, the cell factory *S. cerevisiae*, consequent to the intracellular expression of recombinant EgtA.

## 2. Results

### 2.1. EgtA Expression in Saccharomyces Cerevisiae

**Figure 1:**
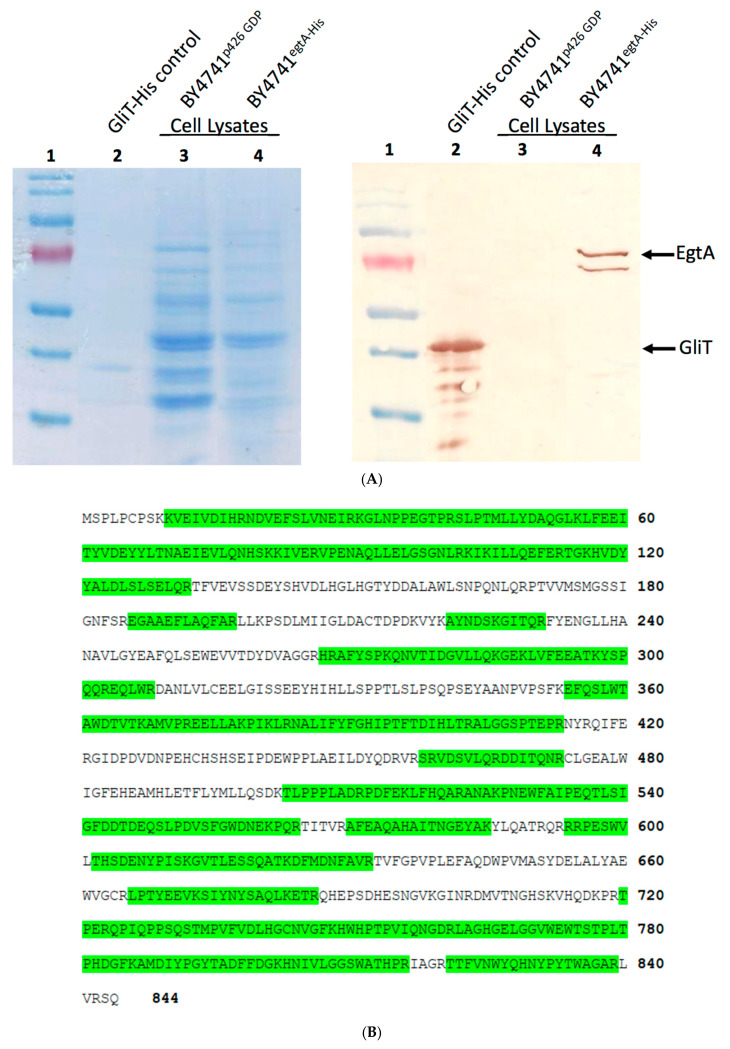
(**A**) Western Blot confirming the expression of His-tagged EgtA in BY4741: Lane 1 = pre-stained protein ladder 10–170 kDa; Lane 2 = positive control recombinant GliT His-tag (36 kDa); Lane 3 = negative control BY4741^p426 GDP-^lysate; Lane 4 = BY4741^egtA-His^ lysate. Western Blot visualised by 3,3′-diaminobenzidine; (**B**) EgtA identified from protein MS analysis of *S. cerevisiae* BY4741*^egtA-His^* cell lysate. EgtA was confirmed to be produced by BY474*^egt-^*^His^ with 61% sequence coverage. Peptides from near the N- and C-termini are present and highlighted in green, indicating that EgtA was intact upon expression, although a smaller probably truncated protein can also be observed.

The plasmid DNA of p*426 GPD*-*egtA^His-tag^* was purified from recombinant *E. coli* and transformed into *S. cerevisiae* strain BY4741 and selected by its ability to grow on –URA SC media. Colony PCR confirmed that four of the five transformants contained *p426 GPD-egtA^His-tag^*, engineered to contain an N-terminal His_6_ tag (Supplementary Figure S1). SDS–PAGE and Western Blot analysis confirmed the expression of EgtA in recombinant *S. cerevisiae* (Figure 1A). Following band excision, corresponding to His-tagged EgtA, and trypsinisation, protein mass spectrometry using the Q-Exactive Orbitrap LC–MS system (Thermo Scientific) identified 69 peptides derived from EgtA corresponding to 61% sequence coverage (Proteome Discover score: 756). This unambiguously confirmed the intact, recombinant EgtA expression and presence in *S. cerevisiae* (Figure 1B).

### 2.2. Intracellular Ergothioneine Production in Recombinant S. cerevisiae–EgtA Using LC–MS Analysis

**Figure 2:**
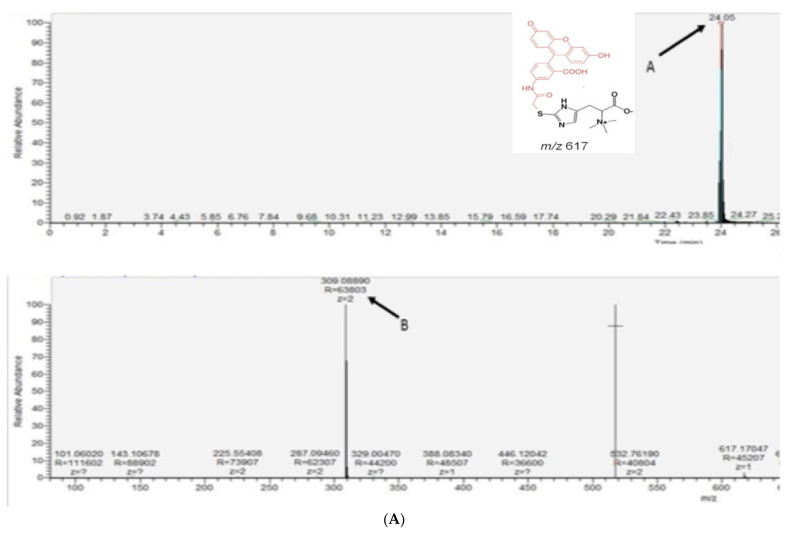

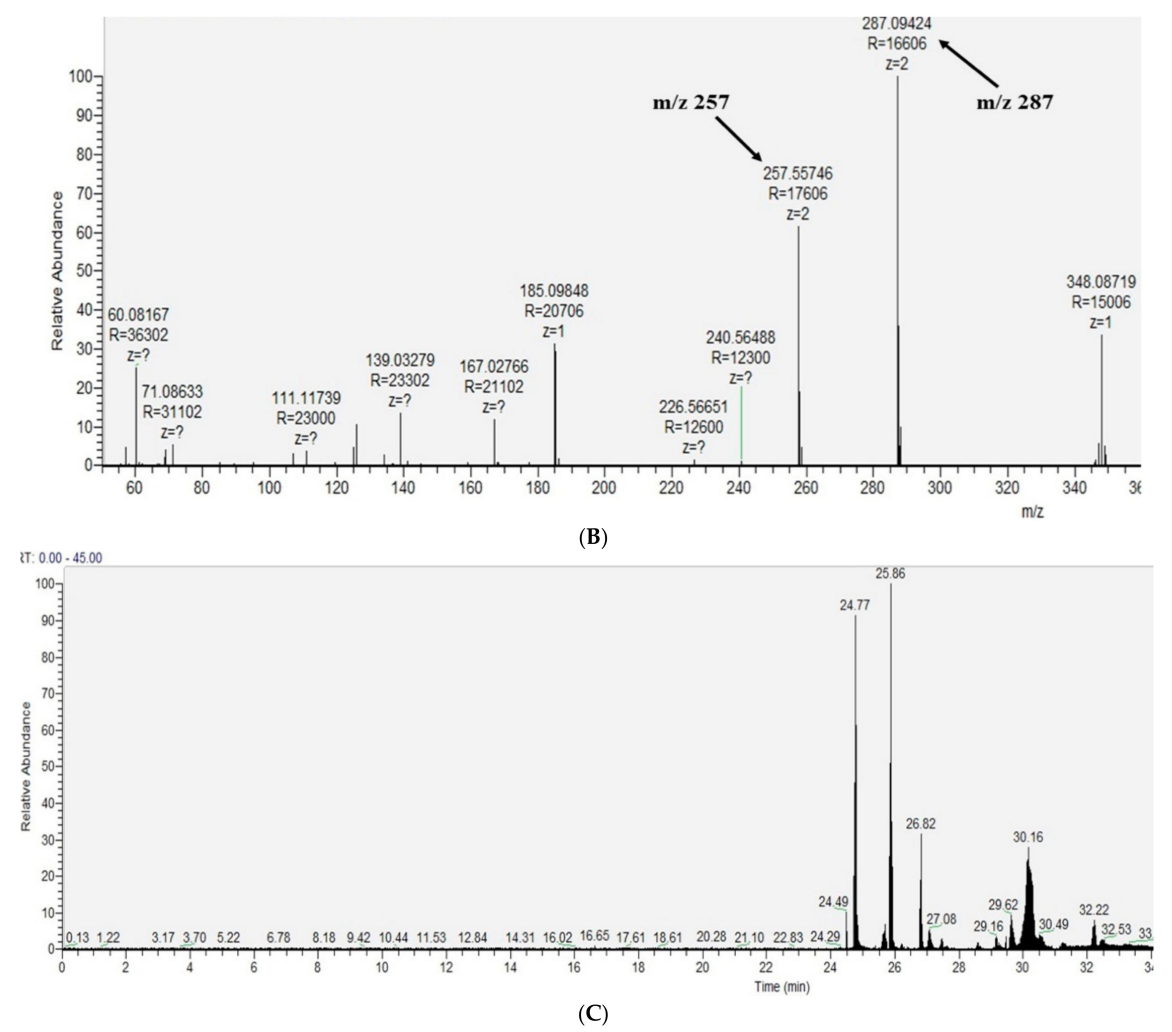
(**A**) Alkylated EGT in BY4741*^egtA-His^*cell lysate. LC–MS of BY4741*^egtA-His^* alkylated cell lysate revealed EGT presence at retention time of 24 min (A), including the double protonated form at *m/z* 309 (B); (**B**) daughter ions *m/z* 287 and *m/z* 257 of *m/z* 309 were identified also in cell lysate of BY4741*^-egtA^*which confirmed the intracellular presence of EGT; (**C**) neither *m/z* 309 or *m/z* 617 was present in the control culture BY4741*^p426-GPD^*, confirming EGT absence.

Ergothioneine was detected in recombinant *S. cerevisiae* lysates following 24 h growth, by the alkylation of lysate EGT with the fluorescent alkylation agent 5′-Iodoacetamidofluorescein (5′-IAF) [6] and subsequent LC–MS analysis. Specifically, the alkylated EGT was identified using its calculated *m/z* and fragmentation pattern, identified by Gallagher et al., comprising *m/z* 617 (M + H)^+^ and *m/z* 309 (M + H)^2+^ when singly and double protonated, respectively. Daughter ions, *m/z* 573 and *m/z* 514, were also detected by LC–MS, along with the double protonated versions of *m/z* 287 and *m/z* 257, respectively [6]. The full scan mass spectra identified alkylated EGT as the double protonated form of the molecular ion *m/z* 309 at a retention time of 24 min in recombinant *S. cerevisiae*–EgtA cell lysates (post-alkylation) (Figure 2A). LC–MS further revealed *m/z* 309 and daughter ions *m/z* 257 and 287, unambiguously confirming the presence of alkylated EGT (Figure 2B). No alkylated EGT ions were detected following a detailed LC–MS analysis of lysates from the empty-vector negative control transformant *S. cerevisiae* BY4741*^p426-GPD^* (Figure 2C).

### 2.3. Extracellular Ergothioneine Production in Recombinant S. cerevisiae–EgtA Using LC–MS Analysis

**Figure 3:**
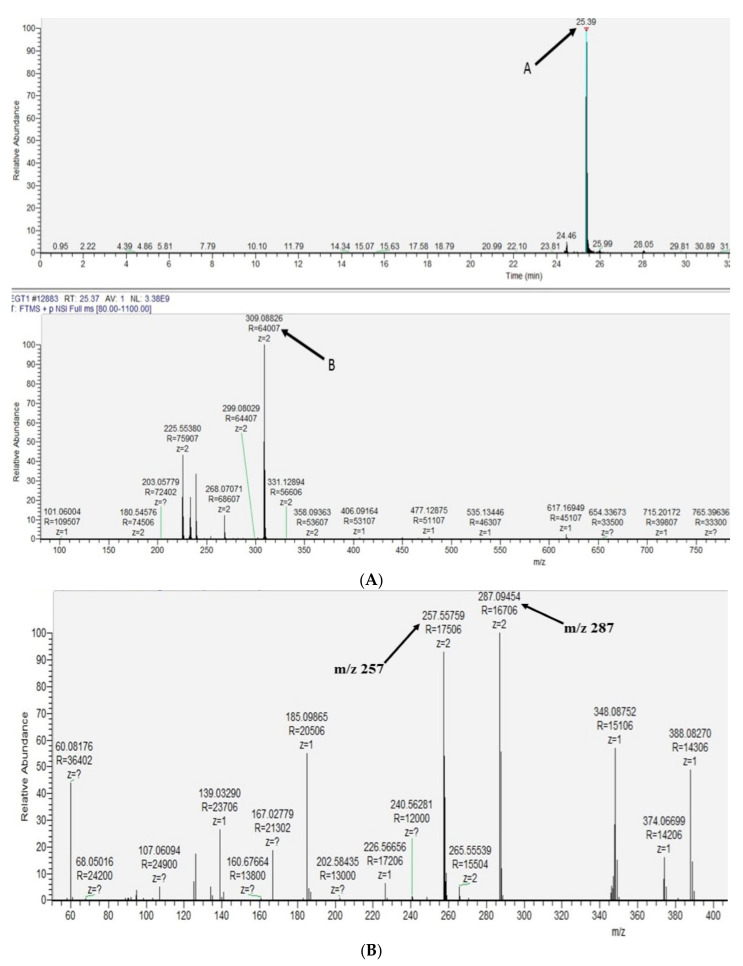
(**A**) The double protonated form of alkylated EGT (M + H)^2+^ = *m/z* 309 was identified in BY4741*^egtA^* culture supernatant at retention time of 25.39 min (A); (**B**) MS^2^ of *m/z* 309 yielded the expected daughter ions of *m/z* 309 (**B**), confirming extracellular alkylated EGT presence.

Extracellular EGT was detected in recombinant *S. cerevisiae*–EgtA culture supernatants following alkylation and LC–MS analysis, as described above. Mass spectral analysis identified the alkylated EGT in the recombinant yeast culture supernatants of BY4741*^egtA-His^*at a retention time of 25.4 min (Figure 3A). The LC–MS analysis of *m/z* 309 revealed the daughter ions *m/z* 257 and 287, again confirming that *m/z* 309 was alkylated EGT (Figure 3B). Neither *m/z* 617 nor *m/z* 309 was detected in the culture supernatants of BY4741*^p426-GPD^* negative control cultures.

### 2.4. Detection of Mainly Extracellular Ergothioneine from Recombinant S. cerevisiae–EgtA Using RP–HPLC Analysis

**Figure 4:**
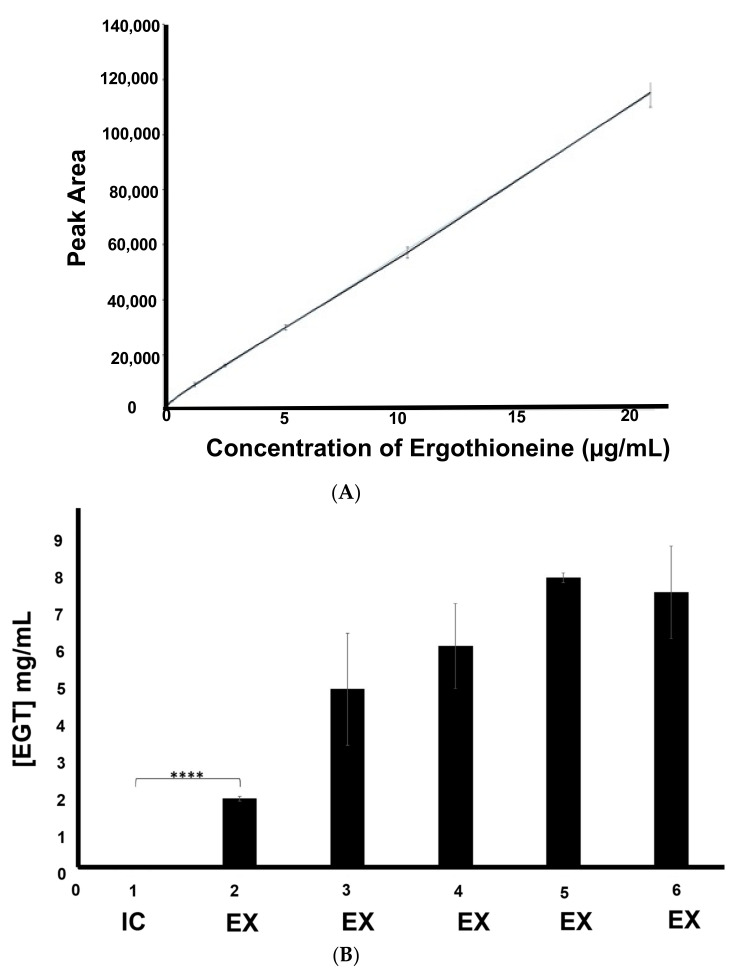

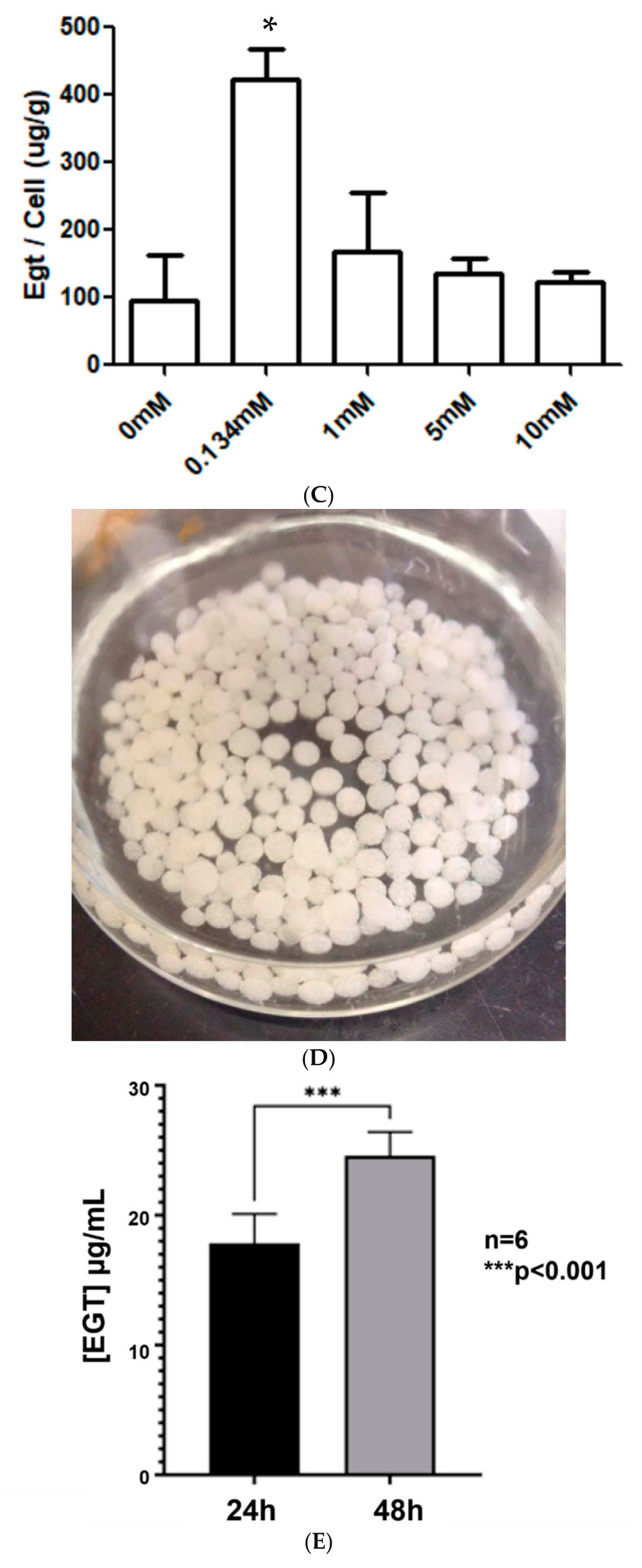

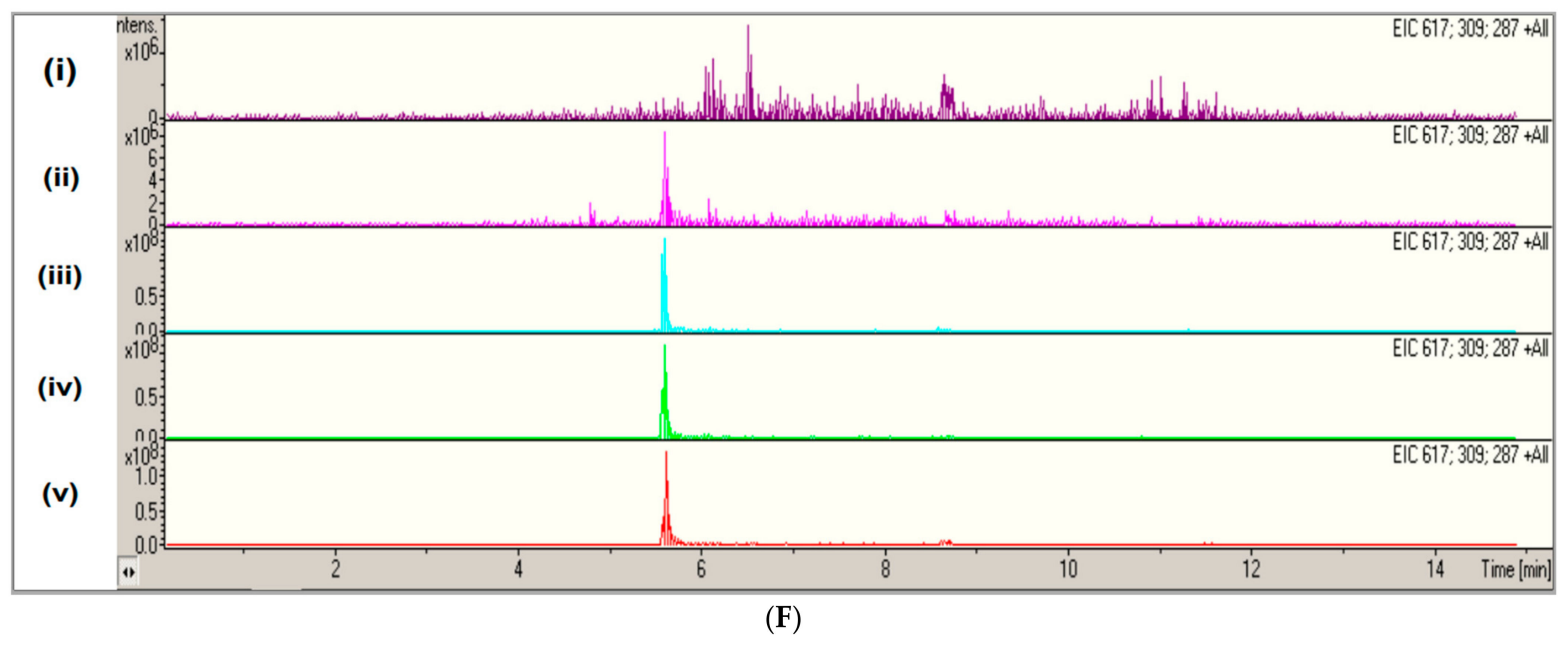
(**A**) Standard curve of labelled EGT. Standard curve generated using 9 EGT standards ranging from 0.08 to 20.83 µg/mL of EGT loaded onto column. Accuracy of standards was assessed by the R^2^ value = 0.9998 indicating a high degree of accuracy; (**B**) quantification of EGT production. Quantity of EGT in BY474*^-egtA^* grown in SC media: 1 = quantity of intracellular (IC) EGT (10.12 µg/L) after 24 h; 2 = quantity of extracellular (EX) EGT (1.91 mg/L) after 24 h, and at significantly **** (*p* ≤ 0.0001) higher amounts; 3 = quantity of extracellular EGT (4.89 mg/L) after 48 h; 4 = quantity of extracellular EGT (6.07 mg/L) after 72 h; 5 = quantity of extracellular EGT (7.93 mg/L) after 96 h; 6 = quantity of extracellular EGT (7.53 mg/L) after 120 h; (**C**) L-Met availability influences EGT production in recombinant *S. cerevisiae*. Amount of secreted EGT produced per weight of cells (μg/g) in BY4741*^egtA-His^* supernatants grown at a range of L-Met concentrations. Cultures grown at 0.134 mM L-Met show a significant * (*p* < 0.05) increase in EGT produced per gram of cells versus cultures grown at all other concentrations of L-Met; (**D**,**E**) EGT is released from immobilised recombinant *S. cerevisiae*–EgtA at 24 h and at significantly *** (*p* < 0.001) higher amounts at 48 h; (**F**) stacked EIC chromatograms following LC–MS analysis of (**i**) negative control, (**ii**) positive control (EGT (5 µg/mL), (**iii**) sample number 1 (**iv**) sample number 4, and (**v**) sample number 6. The peak visible at RT 5.6 min in samples (**ii**)–(**v**), inclusive, is EGT.

The alkylation of EGT standards with 5′-IAF, at a range of concentrations, yielded the standard curve shown in Figure 4A, using the A_442nm_ detection of alkylated EGT (Supplementary Figure S2). Extracellular EGT was identified in the BY4741*^egtA-His^*culture supernatants by comparison with the culture supernatant from the negative control BY4741*^p426-GPD^*(Supplementary Figure S2). Intracellular EGT levels were then investigated, by preparing cultures in triplicate at the time points of 24, 48, and 72 h. Although EGT was detectable in 24 h cell lysates (Figure 4B), no EGT was identified in the cell lysates from 48 h and 72 h cultures. The quantity of EGT in the corresponding culture supernatants of 24 h, 48 h, 72 h, 96 h, and 120 h BY4741*^egtA-His^* cultures was also calculated against the standard curve (Figure 4A) and revealed that the levels of extracellular EGT were significantly higher at 24 h than those of the intracellular EGT (*p* < 0.0001). The quantity of EGT in the culture supernatants of BY4741*^egtA-His^* rose at subsequent time points of 48 h, 72 h, and peaked at 96 h with 7.93 mg/L of EGT detected (Figure 4B). This indicates that the majority of EGT is either effluxed or leaking from recombinant *S. cerevisiae*–EgtA. Subsequent analysis revealed that the relatively low levels of L-Met (0.134 mM) resulted in the significant elevation of EGT biosynthesis and secretion (*p* < 0.05) at 96 h compared with no and 1–10 mM additional L-Met, respectively (Figure 4C). The recombinant *S. cerevisiae*–EgtA was also immobilised in alginate beads and shown to produce extracellular EGT (25 ± 2 µg/L) following incubation up to 48 h at 30 °C (Figure 4D,E), as confirmed via LC–MS analysis (Figure 4E,F).

### 2.5. Co-Expression of egtA with the egtB Candidate AFUA_2G13295 Decreases the Level of EGT in S. cerevisiae

The *egtB* candidates AFUA_2G13295 (*egt2a*) and AFUA_3G14240 (*egt2b*) were identified based on sequence homology compared with the known cysteine desulfurases of the EGT biosynthetic pathway. *Egt2a* is a likely homologue of the cysteine desulfurase gene SPBC660.12c (*egt2* in *Schizosaccharomyces pombe* [17]), with 29% identity. Egt2a shares homology with *N. crassa* Egt2 (45% identity) [3] and mycobacterial EgtE. *Egt2b*, a mitochondrial cysteine desulfurase and a homologue of *nfs1* found in *S. cerevisiae*, was considered the enzyme most likely to participate in EGT biosynthesis in *S. cerevisiae*.

**Figure 5:**
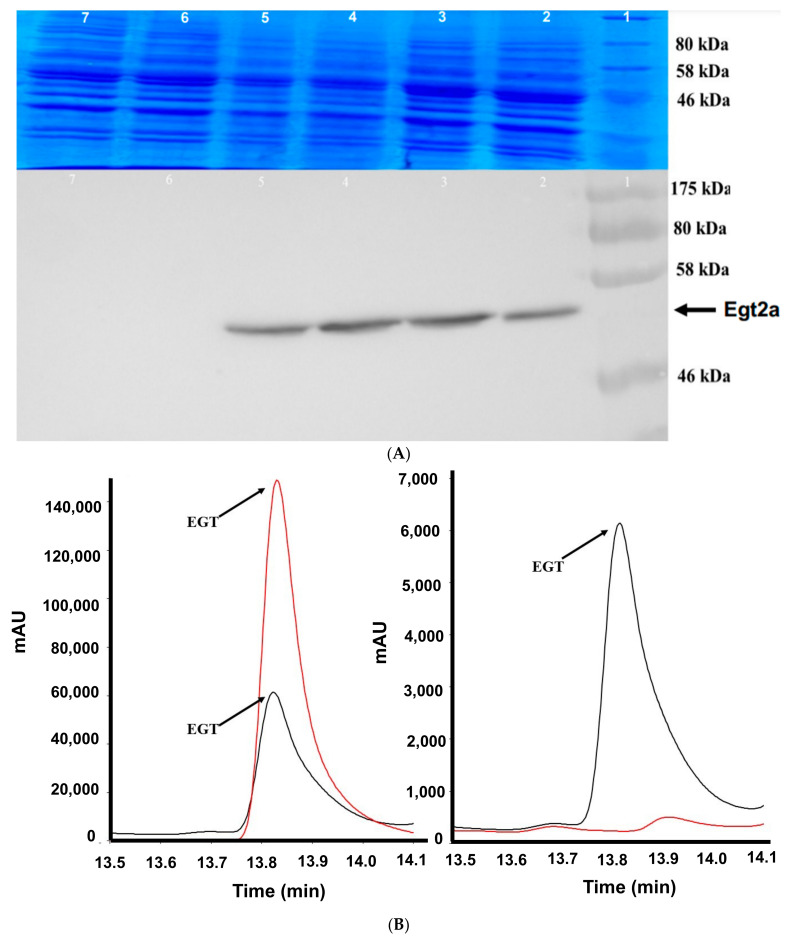

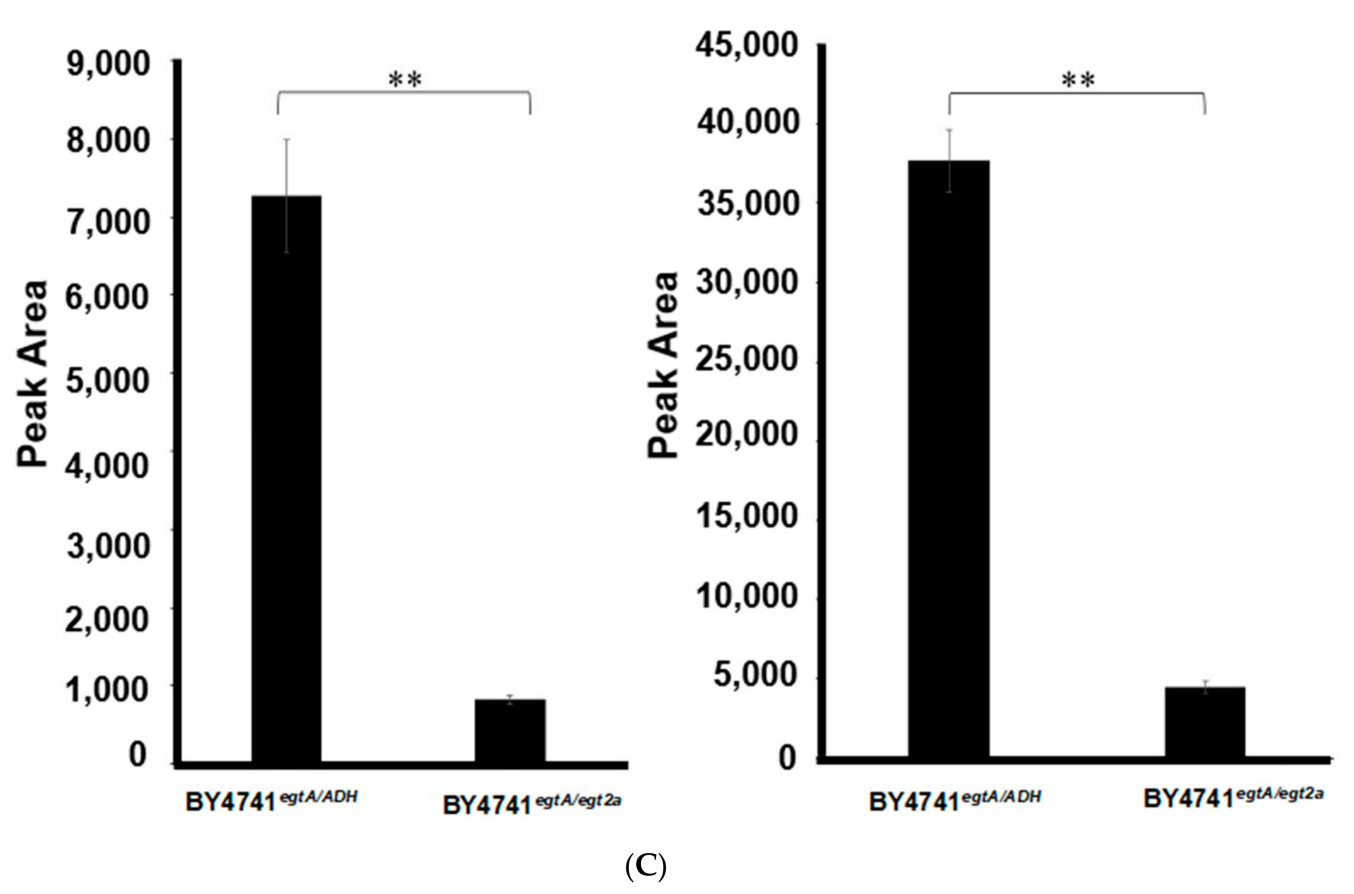
(**A**) Western Blot confirmation of Egt2a expression in BY4741*^egt2a-^*^His^: Lane 1 = protein ladder 175–177 kDa; Lanes 2–5 = independent samples of His-tagged Egt2a protein (50 kDa) from *S. cerevisiae* BY474*^egt2a-^*^His^; Lanes 6–7 = negative control BY4741*^egt2a^*cell lysate; (**B**) EGT production in BY4741*^egtAegt2a^*is confirmed. Left panel shows EGT in BY4741*^egtAegt2a^*(retention time: 13.84 min (black) compared with the EGT standard 13.85 min (red)). Right panel shows EGT in BY4741*^egtAegt2a^* at a retention time of 13.84 min (black) compared with the BY4741*^p426-GPD p423 ADH^* where EGT is absent; (**C**) release of significantly less extracellular EGT from BY4741*^egtAegt2a^* than from BY4741*^egtAp423-ADH^*. Left panel shows culture supernatant EGT peak areas after 48 h and show significantly higher levels [**] (*p* = 0.0063) of EGT in BY4741*^egtAp423 ADH^*than in BY4741*^egtAegt2a^*. Right panel reveals peak areas from EGT supernatant at 72 h which show significantly higher levels [**] (*p* = 0.0018) of EGT in BY4741*^egtAp423 ADH^*than in BY4741*^egtAegt2a^*.

Post-RNA purification, *egt2a* and *egt2b* were PCR-amplified from *A. fumigatus* cDNA using High Fidelity Taq to ensure sequence integrity. The primer set egt2a F and egt2a R amplified *egt2a* (Supplementary Figure S3), and the primer set egt2b F and egt2b R was used for AFUA_3G14240 (Supplementary Figure S3). Vector *p423-ADH* (Supplementary Information) was also digested prior to ligation with restriction enzymes *Spe1* and *EcoR1* for 3 h at 37 °C, followed by agarose gel electrophoresis (Supplementary Figure S3). Plasmids *p423-ADH-egt2a* and *p423-ADH-egt2b*, purified from recombinant *E. coli*, were then individually transformed into the yeast strain BY4741*^egtA^*, and an empty *p423-ADH* plasmid was also transformed into BY4741*^egtA^*. The growth ability on –URA-HIS SC media was determined to select the transformants, followed by colony PCR to assure successful transformation (Supplementary Figure S3). Although *Egt2a* was transcribed and expressed by BY4741*^egt2a^*^-His^ (Figure 5A), no production of Egt2b was detected, using either N- or C-terminal His_6_ tag. An examination of the peak areas facilitated comparative EGT production levels in culture supernatants, and although EGT was produced in BY4741*^egtAegt2a^* (Figure 5B), there was significantly less extracellular EGT detected in BY4741*^egtAegt2a^*than in BY4741*^egtAp423 ADH^* at 48 h (*p* = 0.0063) and 72 h (*p* = 0.0018) (Figure 5C). This led to the conclusion that Egt2a may not be involved in EGT biosynthesis or else may be interfering with EGT production in recombinant yeast.

### 2.6. In Silico Modelling of A. fumigatus EgtA Structure Predicts a Didomain Structure with Opposed Active Sites

**Figure 6:**
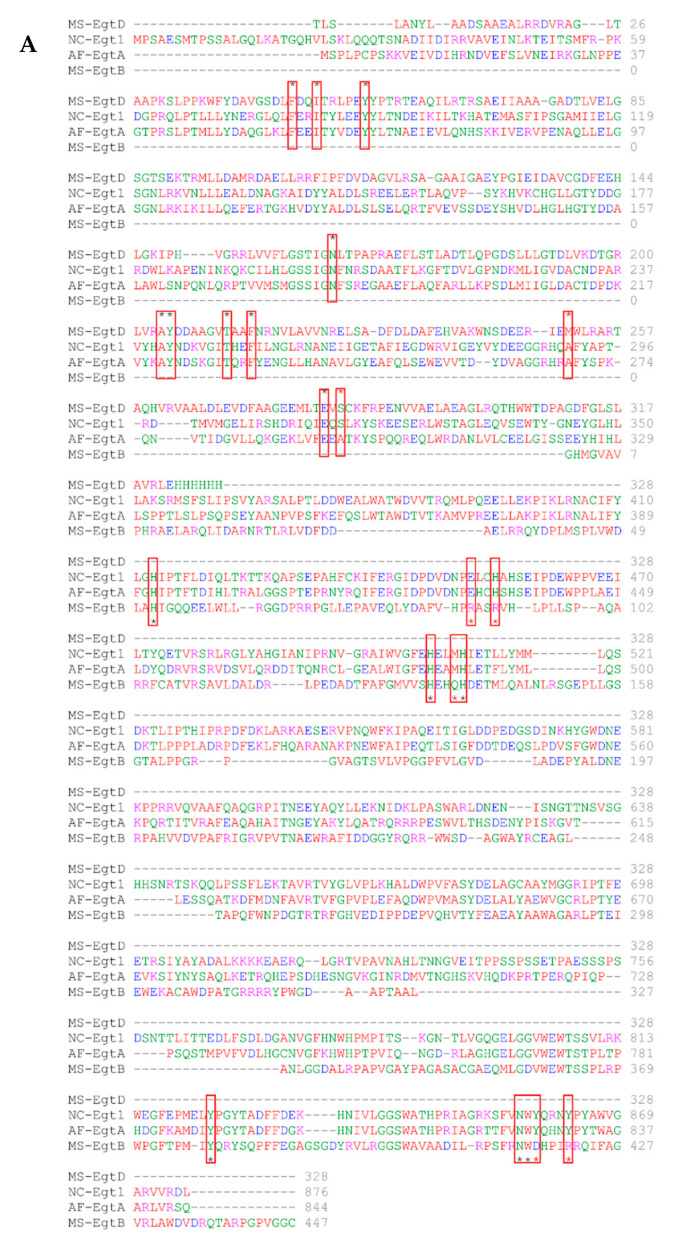

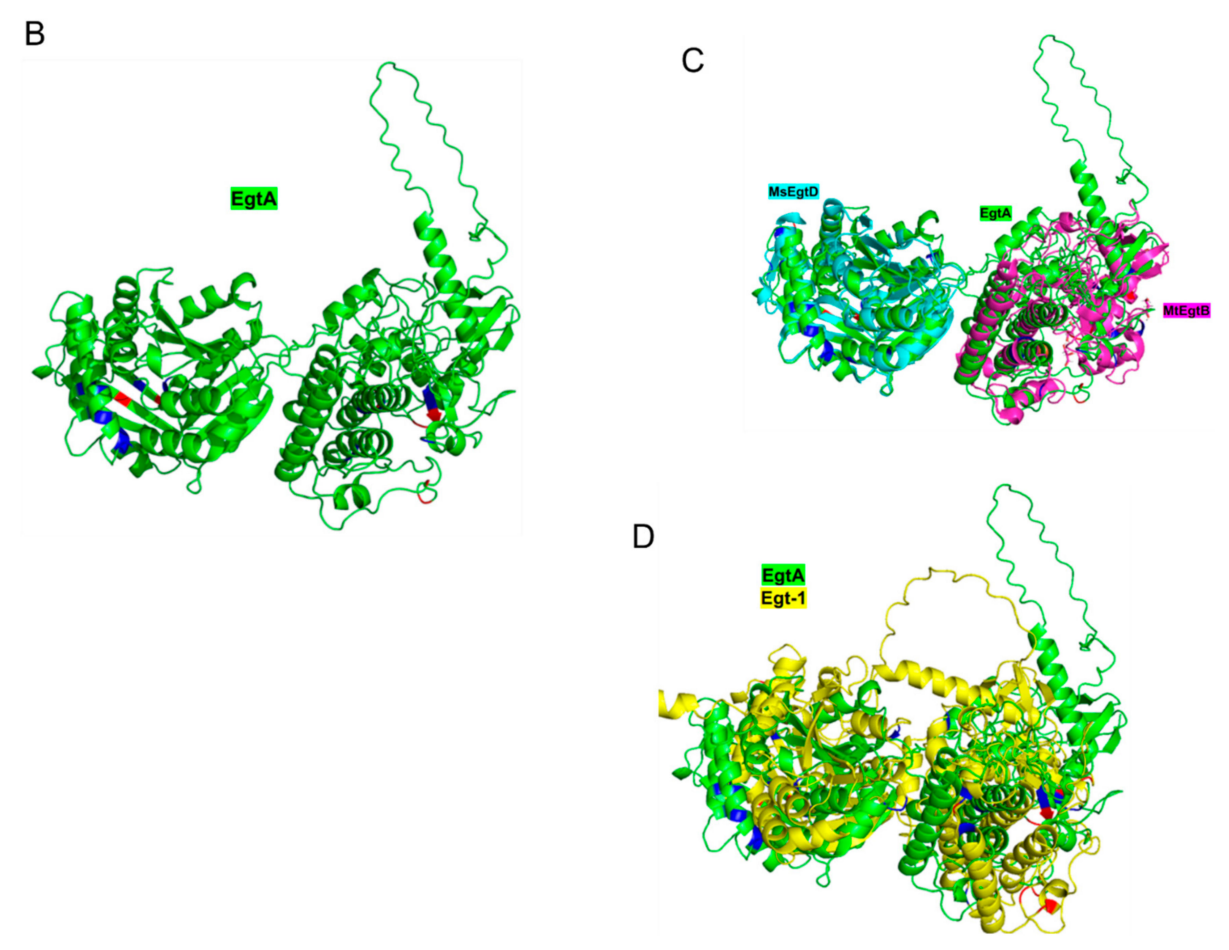
(**A**) Sequence alignment of bacterial and fungal ergothioneine biosynthetic enzymes, showing relevant active site amino acids (red boxes); (**B**) AlphaFold prediction of *A. fumigatus* EgtA 3D structure showing the bidomain enzyme arrangement, with active sites on opposing sites of the monomer (green); (**C**) MsEgtD methyltransferase (magenta) and MtEgtB sulphoxide synthase (pink), respectively, overlaid on *A. fumigatus* EgtA 3D structure; (**D**) the 3D structure of NcEgt-1 (yellow), predicted by AlphaFold, overlaid on the *A. fumigatus* EgtA 3D structure.

Figure 6A shows a multiple sequence alignment of EgtA, NcEgt-1, and bacterial copies of MtEgtB and MsEgtD, with bacterial active site residues highlighted in red boxes. Of the 11 known active sites in MsEgtD, 9 were conserved in both EgtA and NcEgt-1, while only 6 of the 11 active sites of MtEgtB were conserved. Figure 6B shows the 3D structure of EgtA, as predicted by AlphaFold, and reveals a didomain arrangement of the enzyme, as well as the predicted positioning of the D domain (methyltransferase) and B domain (sulphoxide synthase) active sites (red and blue colours) on the opposite sides of EgtA. AlphaFold uses the predicted local distance test (pLDDT) to estimate a per-residue confidence metric. The pLDDT indicates how well a prediction would agree with an experimental structure based on a local distance difference test [35]. A pLDDT > 90 is considered a high accuracy cut-off for AlphaFold predictions. The predicted EgtA structure achieved a mean score of 89.40 (median 94.39), indicating a high confidence prediction. A large sequence loop (E689-Q731), devoid of the predicted structure, was also apparent for EgtA (pLDDT = 32.03, median = 30.08) (Figure 6B), and we speculate this region may play a role in thioether bond cleavage to generate the free thiol on EGT. As can be seen in Figure 6C, the actual crystal structures of MsEgtD and MtEgtB map onto the predicted EgtA structure, and using the global superposition metric template modelling score (TM-score), they achieved a high score of 0.84 and 0.67, respectively (Table 1), thus providing further confidence in the accuracy of our in silico predictions. It is interesting that the bacterial sulphoxide synthase domain and the B domain of EgtA showed less overlap (TM = 0.67) than the corresponding methyltransferase (D) domains (TM = 0.84). Figure 6D shows the overlay of the predicted *A. fumigatus* EgtA and NcEgt-1 3D structures. The predicted NcEgt-1 structure had a mean pLDDT score of 84.387 (median = 92.77), indicating a relatively high accuracy prediction. The TM-score between both predicted fungal structures (EgtA vs. isNcEgt-1) was lower than expected (0.47), and overall, they only shared 29% sequence identity (41% sequence similarity), comparable identity/similarity, as observed between the comparisons of EgtA vs. MsEgtD and EgtA vs. MtEgtB, respectively (Table 1). Like EgtA, NcEgt-1 also appeared to have a disordered region in the B domain (R718-I763, pLDDT = 31.42, median = 26.76), albeit predicted to be less perpendicular and closer to the D domain than EgtA. It is notable that NcEgt-1 appeared incapable of EGT biosynthesis, while EgtA may include the necessary enzymatic armamentarium to make EGT.

## 3. Discussion

Herein, we demonstrated that *A. fumigatus* EgtA expression in *S. cerevisiae* results in the biosynthesis and secretion of EGT in a time-dependent manner. EGT does not accumulate within *S. cerevisiae* and is secreted from both free and alginate-bead immobilised recombinant *S. cerevisiae*–EgtA in fluidised-bed-type fermentation systems. For the first time, the 3D structures of *A. fumigatus* EgtA and *N. crassa* Egt-1 were confidently predicted using AlphaFold and revealed similar, though non-identical, structures (TM = 0.47). Both predicted structures have distinct methyltransferase and sulphoxide synthase domains, where the substrate binding sites are located on the opposite sides of the enzyme. We speculate that either a thiolase activity resides within EgtA, or that *S. cerevisiae* encodes a cryptic thiolase activity which enables EGT formation from the hercynine-Cys intermediate formed by the B domain (Figure 6B) of EgtA. This remains an open and interesting question. EgtA primary sequence does not have a traditional thiolase PFAM domain (Thiolase_N PF00108 or C PF02803) or a predicted thiolase domain (Ecpred), and the enzyme will be screened for such activity in future work. If *S. cerevisiae* does encode cryptic thiolase activity, the question arises as to why this was not detected in other studies, and if such activity does exist, are there perhaps very specific conditions required for its detection?

This work represents the first demonstration that a single gene, *A. fumigatus egtA*, can encode EGT biosynthesis upon recombinant expression. The demonstration of recombinant EgtA enzyme presence through Western blot and LC–MS analysis in the chosen expression system, *S. cerevisiae*, is also unique. To our knowledge, no previous study describing EGT production has simultaneously shown biosynthetic enzyme(s) presence, although SDS–PAGE was carried out in one study to reveal recombinant protein overexpression [33]. Our observation of both intact and partially processed recombinant EgtA in *S. cerevisiae* is suggestive of a C-terminal processing event since the His_6_ tag used for detection was N-terminally located. Importantly, this does not appear to affect the detectable EGT production. While the mechanism by which recombinant EgtA was additionally observed at a slightly lower molecular mass is unclear, if the functionality is altered in vivo through this degradation or premature translation termination, there is still an adequate amount of functional EgtA in the cell to result in EGT production.

EGT is directly detectable using LC–MS; however, specialised LC columns are required due to the polarity of the molecule. We previously demonstrated that EGT alkylation via 5′-iodoacetamidofluorescein resulted in stoichiometric conversion to yield a product of *m/z* [M+H]^+^ 617.3, detectable via LC–MS and DAD/FLD using conventional chromatographic approaches [6]. This strategy was also independently and successfully developed for plasma EGT detection by others [36]. Herein, we further deployed this bioconjugation approach for EGT detection from recombinant *S. cerevisiae,* by both parent and daughter ion derivatised EGT detection, only in the cells transformed with *egtA*. We further revealed via quantitative RP–HPLC that, within 24 h, almost all the EGT was secreted, did not accumulate intracellularly, and that secretion continued up to 96 h. The maximum concentration of extracellular EGT produced was 7.53 mg/L, which is somewhat less than that produced (11.08–20.16 mg/L) by the co-expression of *Grifola fondosa egt-1* and *-2* [37] in yeast. However, it is substantially lower than that impressively achieved in an alternative *S. cerevisiae* system (106.2 mg/L) employing two *egt*-related genes (*N. crassa egt-1* and *Claviceps purpurea egt-2*) plus additional host cell genetic modifications [38] and an optimised fed-batch fermentation system. Conversely, the analysis of these two systems revealed only 24% and no EGT secretion, respectively, in contrast to our deployment of EgtA only, in which all EGT was, and continued to be, effectively released after 24 h fermentation up to 96 h. We also observed that the low concentrations of Met could enhance EGT production and that the recombinant *S. cerevisiae* immobilised in alginate beads, in a fluidised-bed mini-fermenter arrangement, could secrete EGT. Taken together, these observations suggest that future work on fermentation systems and conditions could further enhance the overall productivity of the use of recombinant *S. cerevisiae*–EgtA for EGT production, by maximising the expression system through media optimisation, vector choice (e.g., inducible promoter system), and strain selection (e.g., protease deficient system), and a thorough time course to assess fluctuations in production levels. We speculate that, while EgtA expression alone in *S. cerevisiae* facilitates EGT production, its co-expression with *Claviceps purpurea egt-2* or other thiolase-encoding genes may further enhance production yield. Production bottlenecks may also be overcome by extensive strain engineering to ensure both sufficient amino acid biosynthetic precursors and the absence of any attenuation of EGT efflux from cells.

Although we considered that EGT production by recombinant *S. cerevisiae* could confer tolerance to yeast against oxidative stress or ethanol, as shown in Supplementary Figures S4 and S5, we found no significant differences in the growth of EGT permissive or non-permissive strains when tested up to 2 mM H_2_O_2_ or 8% (*v*/*v*) ethanol. We conclude that this is due to the observed EGT secretion from recombinant *S. cerevisiae* cells, so intracellular levels never accumulate to confer protection against chemical stressors. This contrasts with the situation in *A. fumigatus*, where no EGT secretion was observed, and resistance against the high concentrations of H_2_O_2_ was observed in wild-type but not *A. fumigatus* Δ*egtA* [2]. Intracellular EGT production also contributes to *Mycobacterium tuberculosis* virulence [7]. Thus, EGT secretion may not always be optimal for biotechnological applications, whereas intracellular accumulation may confer chemoresistance phenotypes associated with improved growth, and potentially, improved cell factory productivity.

Although effectively all other studies which have addressed recombinant EGT production observed yield improvements when multiple bacterial or fungal EGT-associated genes were used for cell factory transformation (Supplementary Table S1), we observed significant diminution of EGT production upon a confirmed expression of a putative cysteine desulfurase (*Afegt-2*) simultaneously with *A. fumigatus egtA*. The reason for this is unclear; however, it could be due to the reduced EgtA abundance upon dual protein expression or the possible competition between cysteine desulfurase (Afegt-2) and any putative thiolase activity within EgtA for EGT, or a related metabolite, formation from the hercynine-Cys intermediate. Relevantly, the recombinant expression of *N. crassa* Egt-1 was insufficient for EGT production [31,38], and a comparative analysis of AlphaFold predicted crystal structures of EgtA and NcEgt-1 revealed differences in the B (sulphoxide synthase) domain of both enzymes. It merits some speculation that this domain in EgtA may encode a cryptic cysteine desulfurase activity capable of conferring the full EGT biosynthetic potential on EgtA. Indeed, it has recently been reported that *Trichoderma reesei* Egt1 expression in *E. coli* may facilitate low-level EGT production [33]. The only plausible alternative is that an endogenous cysteine desulfurase activity resides in *S. cerevisiae* BY4741, which is active in our system. However, this begs the question as to why the published studies using *S. cerevisiae* to produce EGT did not detect this activity. Future work will address the issue of cryptic cysteine desulfurase activity in *S. cerevisiae* BY4741.

## 4. Methods

### 4.1. Cloning and Expression of A. fumigatus egtA and Related Genes in S. cerevisiae

All the strains, plasmid vectors, and primers are shown in Table 2 and Table 3 and Supplementary Table S2 [39,40,41]. *EgtA* cDNA was obtained from GenScript (Piscataway, NJ, USA) and blunt-end-cloned into *pUC57*. *EgtA* was then amplified using High Fidelity Taq polymerase to ensure sequence integrity using the primers EgtA F and EgtA R (Supplementary Table S2). A cloning strategy was employed using the restriction sites *Spe*1 and *Xho*1 at the 5′ and 3′ ends, respectively. The PCR product was separated via gel electrophoresis to check for a single band at the correct size of 2.5 kb and ligated into the vector *p426 GPD* (Table 3), transformed initially into *Escherichia coli*. Then, after purification, *p426 GPD-egtA* was isolated and transformed into the *S. cerevisiae* strain BY4741 (Table 2). Successful transformants (*S. cerevisiae* BY4741*^egtA^*) (Table 3) were selected by determining their growth on –URA SC media via colony PCR to confirm successful transformation and that *p426 GPD-egtA* was present. *S. cerevisiae* BY4741*^egtA-^*^His^ (Table 3) was also generated by an identical strategy to yield an N-terminal His_6_ tag on recombinant EgtA. The *egtB* candidates AFUA_2G13295 (*egt2a*) and AFUA_3G14240 (*egt2b*) were selected due to their sequence homology with the known cysteine desulfurases (C–S bond cleavage) used in the EGT biosynthetic pathway. For both these genes, RNA was extracted from the *A. fumigatus* strain AF293, as described, and reverse-transcribed into cDNA. *Egt2a* and *egt2b* were amplified from cDNA via PCR using High Fidelity Taq polymerase, and *egt2a* was amplified using the primer set egt2a F and egt2a R. For AFUA_3G14240, the primer set egt2bF and egt2b R was used (Supplementary Table S2). The cloning of both *egt2a* and *egt2b* used the cloning strategy *Spe*1 and *EcoR*1 restriction sites at the 5′ and 3′ ends, respectively, and were cloned into the vector *p423-ADH* (Table 3). Following plasmid purification after *E. coli* transformation, both purified plasmids (*p423-ADH-egt2a^His-tag^* and *p423-ADH-egt2b^His-tag^*) (Table 3) were separately transformed into the *S. cerevisiae* strain BY4741*^egtA^*, as described above for *egtA*. *S. cerevisiae* BY4741 was also transformed with empty vectors to serve as negative controls for subsequent experiments.

### 4.2. Protein and EGT Extraction from S. cerevisiae

*S. cerevisiae* cells were washed with MilliQ water, and 50 mg cells were transferred to clean tubes, followed by the addition of Cell Lytic^TM^ Yeast cell lysis reagent (900 µL) and 0.5 mm soda lime glass beads (Biospec, Bartlesville, OK, USA) until only a small amount of space remained. DTT (10 mM) was included to aid protein extraction but omitted when the 5′-IAF labelling of EGT was to be undertaken. The tubes were then bead-beaten (Retsch mm 400, Haan, Germany) for 10 s and then chilled on ice for 1 min, followed by 10× repetition prior to centrifugation at 4 °C for 10 min at 13,000× *g*. The supernatants were transferred to pre-chilled microfuge tubes prior to further analysis.

### 4.3. Immunoblot Analysis of Yeast Cell Lysates

Following the recombinant and control *S. cerevisiae* cell lysate separation via SDS–PAGE and subsequent electrotransfer, nitrocellulose membranes were blocked (5% (*w*/*v*) Marvel in PBST) and probed with murine anti-His_6_ monoclonal IgG (Biolegend, San Diego, CA, USA; 1/3000) in 1% (*w*/*v*) Marvel in PBST for 1 h at RT and PBST wash. Horseradish peroxidase-conjugated anti-mouse IgG (Biolegend; 1/3000 as above) was followed by either diaminobenzidine or chemiluminescent detection for His_6_-tagged recombinant protein detection. Chemiluminescence used ECL substrate (Bio-Rad (Hercules, CA, USA) Clarity^™^ Western ECL Blotting Substrate kit), and imaging was performed via G: BOX F3 (SYNGENE) as per the manufacturer’s instructions.

### 4.4. Alkylation of Intracellular and Extracellular EGT

The recombinant *S. cerevisiae* cell lysates were diluted ¼ (30 µL to 120 µL with PBS) followed by the addition of 5′-IAF (10 µL; 3 mg/mL) per sample. Alkylation was allowed to proceed for 45 min in the dark at room temperature, followed by centrifugation at 13,000× *g* for 5 min at 4 °C. The supernatants (85 µL) were each treated with 15 µL of 100% (*w/v*) TCA and incubated at 4 °C for 3 h, followed by centrifugation (13,000× *g*) for 20 min at 4 °C. They were then removed and placed in fresh tubes for RP–HPLC and LC–MS analyses. For the analysis of extracellular EGT, the culture supernatants (500 µL each) were dried under vacuum and then resuspended in 100 µL of 200 mM sodium phosphate pH 7.6 by vigorous pipetting. After centrifugation (13,000× *g*, 5 min, at 4 °C), 50 µL of each resuspended sample was taken, and 10 µL of 5′-IAF (3 mg/mL) was added. The samples were incubated in the dark for 45 min and then centrifuged (13,000× *g*, 5 min, at 4 °C) prior to analysis. The positive EGT control was prepared by adding 10 µL of 3 mg/mL 5′-IAF to EGT (50 µL; 50 µg/mL) in a 200 mM sodium phosphate buffer pH 7.6. For standard curve preparation, EGT (100 µg/mL) was serially diluted ½ to 1.56 µg/mL, followed by the addition of 10 µL of 5′-IAF (3 mg/mL) to each tube containing EGT (50 µL/standard in 200 mM sodium phosphate pH 7.6). The standard reactions were performed through incubation in the dark for 45 min followed by centrifugation (13,000× *g*, 5 min, at 4 °C) prior to analysis. The negative controls were prepared by the addition of 10 µL of 5′-IAF (3 mg/mL) to 50 µL of 200 mM sodium phosphate pH 7.6, followed by incubation as described above.

### 4.5. RP–HPLC and LC–MS Analyses of Alkylated Ergothioneine

The alkylated samples or standards (50 µL each) were placed in HPLC vials, and in all the cases, 30 µL of each was injected onto a Shimadzu Prominence HPLC system, equipped with an Agilent Eclipse XDA-C18 column equilibrated in Solvent A (5% (*v/v*) acetonitrile in 0.1% TFA, DAD (SPD-M20A), and fluorescent detection. The alkylated EGT was detected following gradient elution (5–100% B/25 min) at 442 nm at a flow rate of 1 mL/min. Solvent B was 0.1% (*v/v*) TFA in acetonitrile. For LC–MS, all the samples were diluted 1/100 in 0.1% (*v/v*) formic acid, followed by spin filtration at 13,000× *g* (1 min, RT). The samples (40 µL each) were then transferred to vials prior to mass spectrometric analysis using Q-Exactive LC–MS as previously described [42,43].

### 4.6. Yeast Culture and Immobilised Yeast (IY) Preparation in Alginate Beads

Under sterile conditions, a recombinant *S. cerevisiae* colony was mixed into 5 mL SC media and placed in a shaking incubator (220 rpm, 30 °C) overnight. Culture OD was calculated, and a volume was aseptically added to 50 mL sterile media in a 250 mL conical flask, in order to prepare a culture of starting OD 0.2, which was then placed in the shaking incubator (220 rpm, 30 °C) for 24 h. Subsequently, the yeast cultures of OD 0.2 (final) were prepared and mixed 1:1 with 3% (*w/v*) sodium alginate. Under sterile conditions, the solution was drawn into a 20 mL syringe, and a 4 mm × 19 mm needle was attached. The 1:1 solution was dropped into 20 mL 0.05 M CaCl_2_ at a 45° angle, producing relatively uniform, circular beads. These yeast-alginate beads were allowed to harden in 0.05 M CaCl_2_ for 1 h and were then washed in fresh 0.05 M CaCl_2_ and stored at 4 °C prior to use. The yeast-alginate beads were placed in 20–25 mL media (Figure 4D) in 100 mL sterile conical flasks and incubated at 30 °C for various time periods.

### 4.7. AlphaFold Prediction of Fungal EGT Biosynthetic Enzyme Structure

The sequences for *A. fumigatus* EgtA (XP_755900.1), *N. crassa* Egt-1 (XP_956324.3), *M. smegmatis* EgtD (4PIM_A), and *M. thermoresistibile* EgtB (4X8B_A) were retrieved from NCBI GenBank and aligned using Clustal Omega [44]. Previous work has established the crystal structures of *M. smegmatis* EgtD [45,46,47], and the crystal structures of ergothioneine sulphoxide synthase from *Mycobacterium thermoresistibile* (EgtB_thermo_) and *Candidatus Chloracidobacterium thermophilum* (EgtB*_Cth_*) have also been elucidated [48,49]. In the absence of fungal enzyme structure, AlphaFold [35] was used to predict the 3D structure of both *A. fumigatus* EgtA and *N. crassa* Egt-1. The prediction process has previously been described (PMID: 34265844) and consists of five steps: multiple sequence analysis construction, template search, inference with multiple models, model ranking based on mean pLDDT, and the constrained relaxation of the predicted structures. Once the fungal enzyme structures were available, a comparative 3D structure analysis was undertaken in PyMOL (The PyMOL Molecular Graphics System, Version 2.52, Schrödinger, LLC.), whereby EgtD and B were mapped to EgtA, and the predicted structure of Egt-1 was overlayed on EgtA. The template modelling score (TM-score) [50] between the structures was undertaken using the Pairwise Structure Alignment tool of PDB (https://www.rcsb.org/alignment, accessed on 10 May 2022). The TM-score is a measure of topological similarity between the template and model structures and ranges between 0 and 1, where 1 indicates a perfect match. Scores < 0.2 usually indicate that the proteins are unrelated, while those >0.5 generally have the same protein fold.

## 5. Conclusions

Unlike the previous studies employing multiple expression systems, we showed that it is possible to produce EGT in *S. cerevisiae* by expressing a single gene encoding for the EgtA protein from *A. fumigatus*. This raises the key question of whether an uncharacterised thiolase activity exists within the EgtA protein, or that an as-yet-unidentified cryptic thiolase capability exists within yeast. Our study further highlights baker’s yeast as a plausible alternative to the chemical synthesis of EGT and a potential reliable cell factory for EGT production and one that is worthy of further study and optimisation.

## Figures and Tables

**Table 1:** Pairwise structural alignments of various Egt protein structures.

Comparison	TM-score	SI%	SS%	Length
EgtA vs. Egt-1	0.47	29	41	787
EgtA vs. MsEgtD	0.84	25	46	315
EgtA vs. MtEgtB	0.67	24	35	388
Egt-1 vs. MsEgtD	0.8	25	44	312
Egt-1 vs. MtEgtB	0.66	23	37	380

TM-score: (template modelling score), SI%: (sequence identity %), SS%: (sequence similarity %). Length structurally equivalent residue pairs.

**Table 2:** *S. cerevisiae* and bacterial strains.

Strain	Genotype	Source
BY4741	MATa *his3*Δ 1; *leu2*Δ; *met15*Δ 0; *ura3*Δ 0	Euroscarf
R1158	MATa *his3*Δ 1; *leu2*Δ; *met15*Δ; pNFS1::kanR-tet07-TATA; *ura3*::CMV-tTA	Dharmacon
R1158	MATa *his3*Δ 1; *leu2*Δ; *met15*Δ; pSAH1::kanR-tet07-TATA; *ura3*::CMV-tTA	Dharmacon
Y258	MATa pep4-3; his4-580; ura3-52; leu2-3	Dharmacon
Top 10	*E. coli*	Thermofisher
Xli-blue	*E. coli*	Thermofisher

**Table 3:** Plasmid vectors used in this study.

Plasmid Name	Description	Source
pUC57	*E. coli* expression vector	GenScript
pRS316	Centromeric *Saccharomyces cerevisiae* shuttle vector—*URA3* marker	Sikorski and Hieter [40]
pC210	*SSA1* under control of *SSA2* promoter—*LEU2* marker	Schwimmer and Masison [41]
p413-ADH	Low-copy centromeric plasmid with an *ADH* promoter based on the pRS vector series—*HIS3* marker	Mumberg et al. [39]
p413-GPD	Low-copy centromeric plasmid with a *GPD* promoter based on the pRS vector series—*HIS3* marker	Mumberg et al. [39]
p416-ADH	Low-copy centromeric plasmid with an *ADH* promoter based on the pRS vector series—*URA3* marker	Mumberg et al. [39]
p416-GPD	Low-copy centromeric plasmid with a *GPD* promoter based on the pRS vector series—*URA3* marker	Mumberg et al. [39]
p423-ADH	High-copy 2µ plasmid with an *ADH* promoter based on the pRS vector series—*HIS3* marker	Mumberg et al. [39]
p423-GPD	High-copy 2µ plasmid with a *GPD* promoter based on the pRS vector series—*HIS3* marker	Mumberg et al. [39]
p426-ADH	High-copy 2µ plasmid with an *ADH* promoter based on the pRS vector series—*URA3* marker	Mumberg et al. [39]
p426-GPD	High-copy 2µ plasmid with a *GPD* promoter based on the pRS vector series—*URA3* marker	Mumberg et al. [39]
p426-GPD-egtA	*Aspergillus fumigatus egtA* under control of *GDP* promoter—*URA3* marker	This study
p423-ADH-egt2a	*Aspergillus fumigatus egt2a* under control of *ADH* promoter—*HIS3* marker	This study
p423-ADH-egt2b	*Aspergillus fumigatus egt2b* under control of *ADH* promoter—*HIS3* marker	This study

## Data Availability

All data in both manuscript and Supplementary Data File.

## Supplementary Materials

The following supporting information can be downloaded at: https://www.mdpi.com/article/10.3390/ijms231810832/s1.
