# Supplementary material for: A Single Aspergillus fumigatus Gene Enables Ergothioneine Biosynthesis and Secretion by Saccharomyces cerevisiae
Source: Int J Mol Sci. 2022 Sep 16;23(18):10832. doi: 10.3390/ijms231810832 (PMC9502471; doi:10.3390/ijms231810832)
Supplement: Supplementary file 1 [file ijms-23-10832-s001.zip › Supplementary Data.pdf]

Running Head: Engineered yeast makes ergothioneine.

August 4th version

Supplementary Data File

A Single *Aspergillus fumigatus* Gene Enables Ergothioneine Biosynthesis and Secretion by *Saccharomyces cerevisiae.*

Sean Doyle[1], Daragh D. Cuskelly[1,2], Niall Conlon[1,3], David A. Fitzpatrick[1], Ciara B. Gilmartin[1], Sophia H. Dix[1] and Gary W. Jones[1,4].

[1] Department of Biology, Maynooth University, Maynooth, Co. Kildare, Ireland.

[2] Present Address: Kerry Group, Naas, Co. Kildare, Ireland.

[3] Present Address: Pfizer, Grangecastle, Co. Dublin, Ireland.

[4] Centre for Biomedical Science Research, School of Health, Leeds Beckett University, Leeds LS1 3HE, United Kingdom.

Corresponding author:

Professor Gary W. Jones FRSB

Centre for Biomedical Science Research,

School of Health

Leeds Beckett University,

Leeds LS1 3HE,

United Kingdom.

Email: gary.jones@leedsbeckett.ac.uk

Telephone: +44 (0)113 812 3764

Keywords: Antioxidant; alphafold; cell factory; redox stress; ROS, ergothioneine.

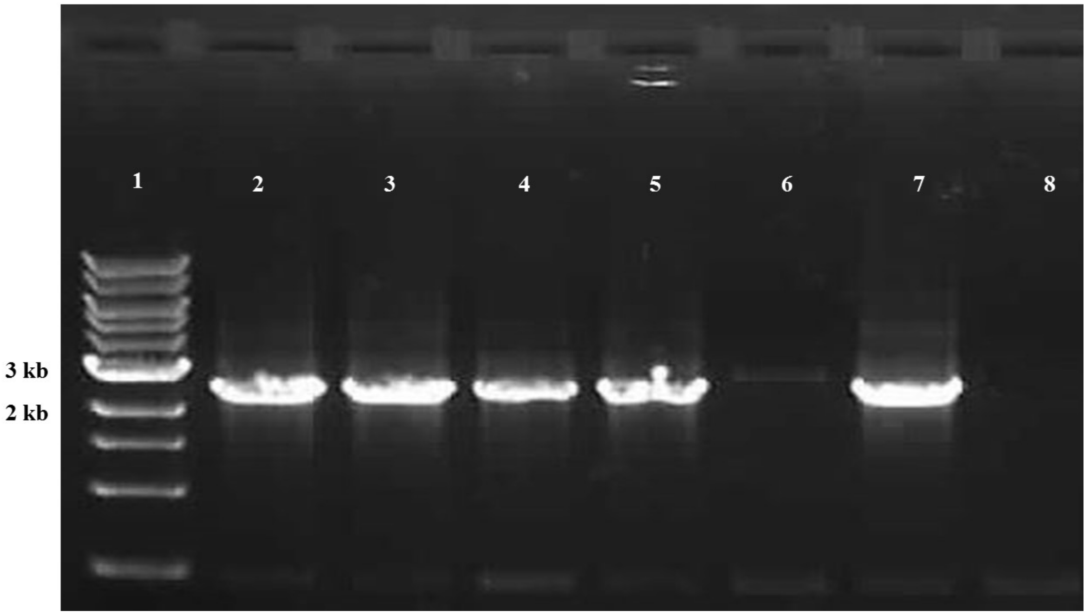

Supplementary Figure S1. Confirmation of *egtA* in BY4741$^{egtA\text{-His}}$ by colony PCR. Lane 1= 10 kb NEB DNA ladder, lanes 2-5= *egtA* in *S. cerevisiae* BY4741$^{egtA\text{- His}}$ (size 2.5 kb) amplified with High Fidelity Taq polymerase, lane 6= BY4741 colony that did not contain *egtA*, lane 7= positive control *p426 GPD-egtA* (size 2.5 kb), lane 8= negative control.

A.

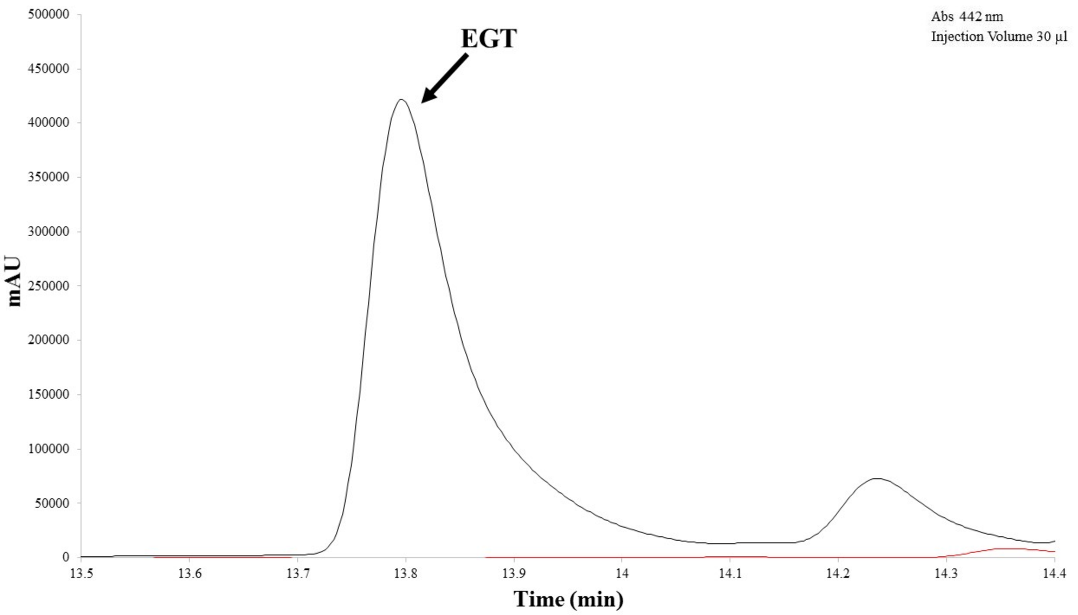

Supplementary Figure S2. Detection of alkylated EGT standard (100 µg/ml) compared to PBS negative control. Alkylated EGT elutes from the stationary phase at a retention time of approximately 13.8 min (black) with no peak in PBS control (red).

B.

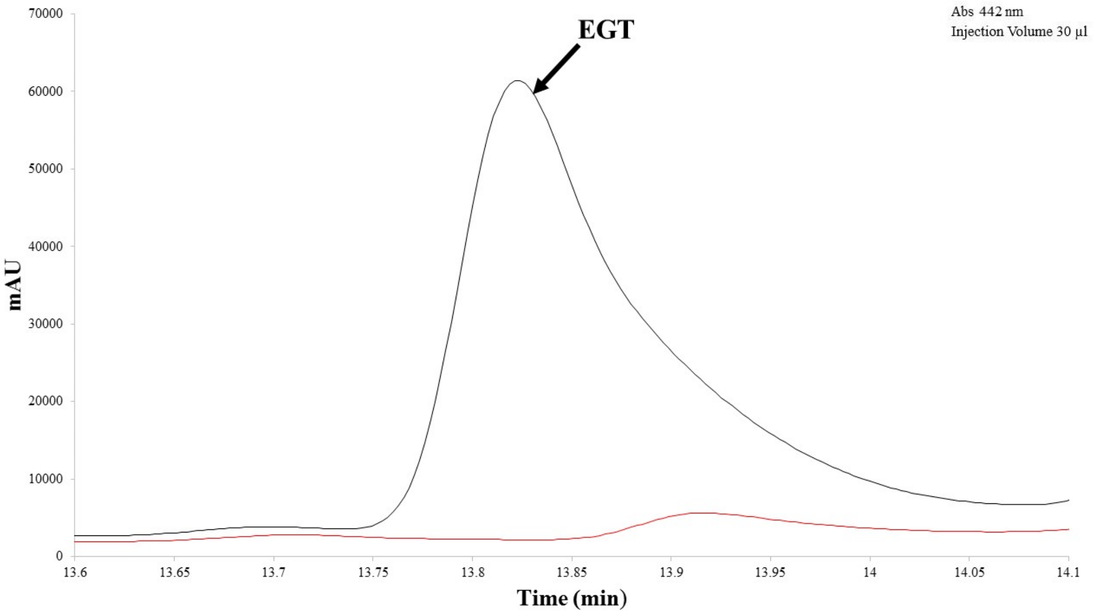

Supplementary Figure S2. Detection of EGT in BY4741$^{egtA\text{-His}}$ in culture supernatants using RP-HPLC analysis. Novel peak identified in alkylated culture supernatants of

BY4741$^{egtA\text{-}His}$ (black) when compared to alkylated culture supernatant from BY4741$^{p426\text{-}GPD}$ (red) matches the retention time of alkylated EGT standard of 13.8 min.

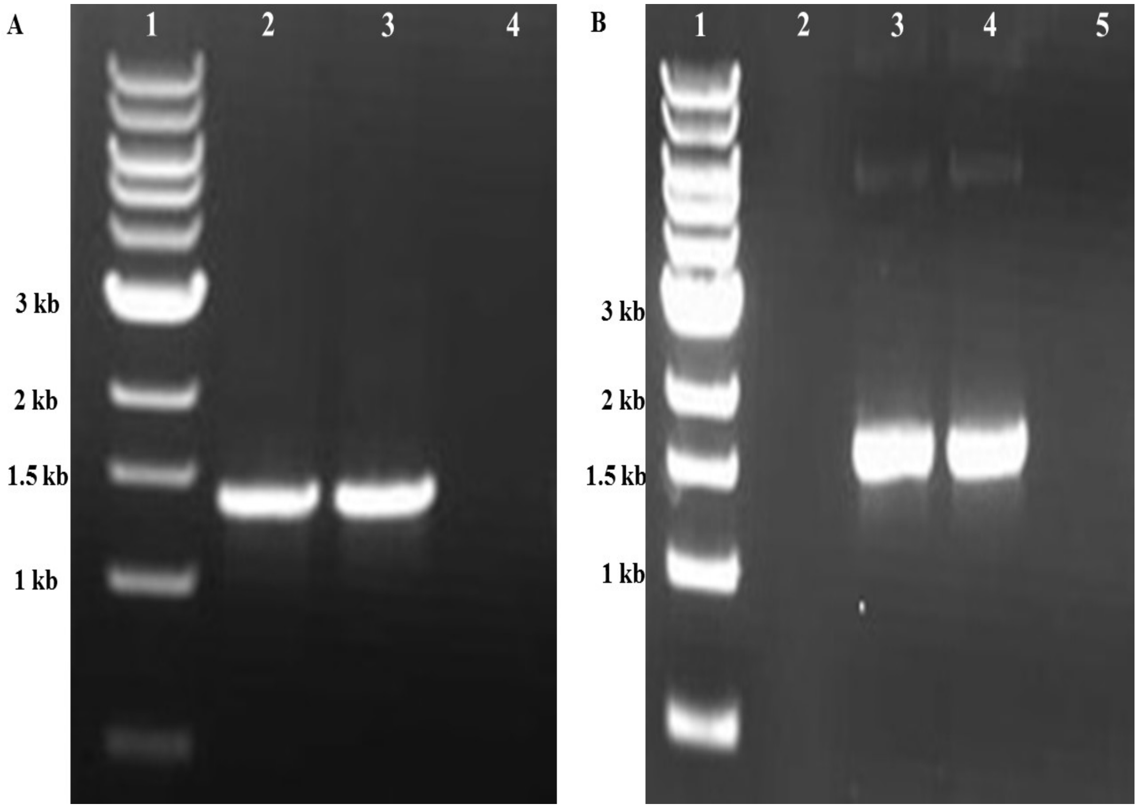

Supplementary Figure S3. A. Amplification of *egt2a* and *egt2b* from AF293 cDNA. (A) Lane 1= 10 kb NEB DNA ladder, lanes 2 & 3= *egt2a* (size 1.3 kb), lane 4= negative control. (B) Lane 1= 10 kb NEB DNA ladder, lane 2= empty, lanes 3 & 4= *egt2b* (size 1.55 kb), lane 5= negative control.

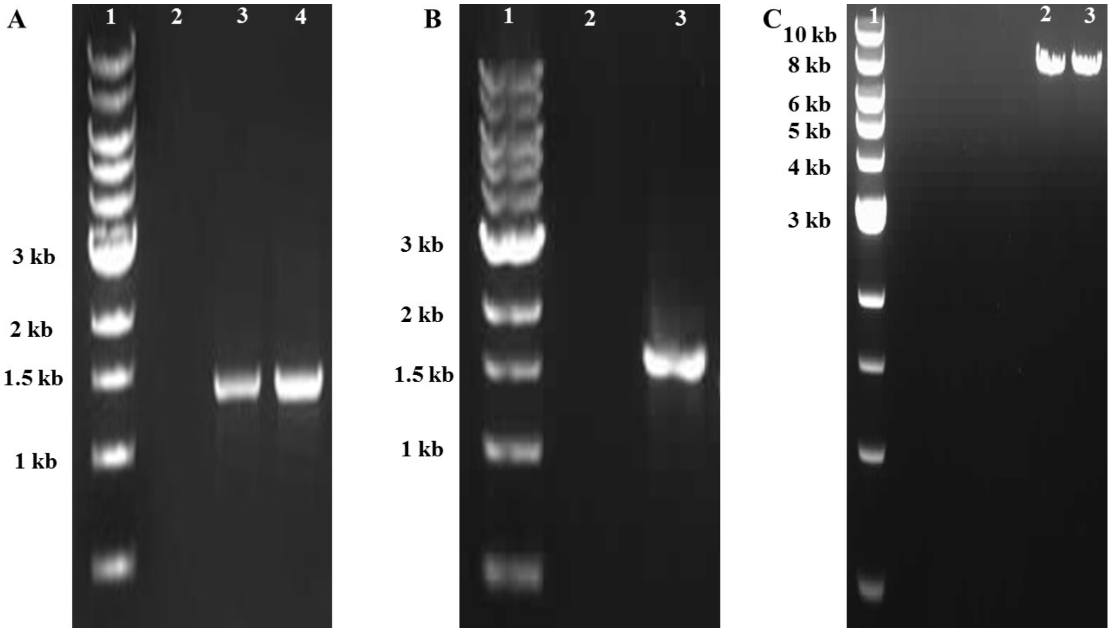

Supplementary Figure S3. B. Digestion of *egt2a*, *egt2b* and *p423 ADH* with the restriction enzymes *Spe1* and *EcoR1*. Lane 1= 10 kb NEB DNA ladder, (A) lane 2= empty, lanes 3 & 4= digest of *egt2a* (size 1.3 kb), (B) Lane 2= empty, lanes 3 = Digest of *egt2b* (size 1.55 kb), (C) Lanes 2 & 3= digest of *p426 GPD* (size 7.6 kb).

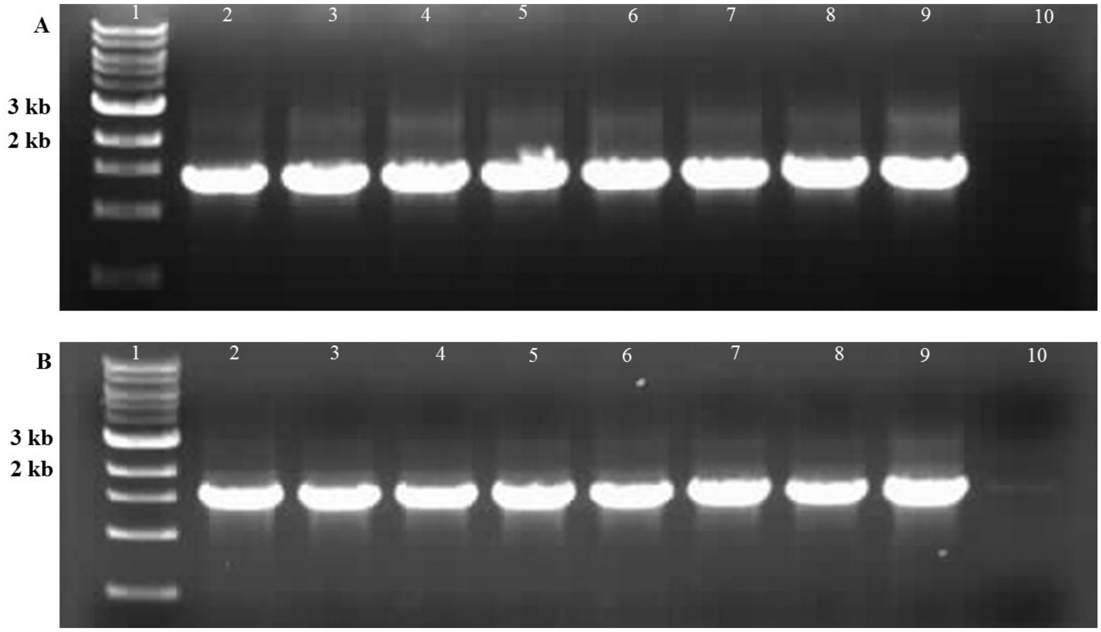

Supplementary Figure S3. C. Successful transformation of *egt2a* and *egt2b* into BY4741$^{p426\text{-}GPD\text{-}egtA}$ confirmed by colony PCR. (A) Lane 1= 10 kb NEB DNA ladder, lanes 2-8 = *egt2a*(size 1.3 kb) from *S. cerevisiae* BY4741^{egtAegt2a} amplified with high fidelity taq, lane 9= positive control *egt2a* (size 1.3 kb) from *p423-ADH-egt2a*, lane 10= negative control. (B) Lane 1= 10 kb NEB DNA ladder, lanes 2-8 = *egt2b* (size 1.55 kb) from *S. cerevisiae* BY4741^{egtAegt2b} amplified with high fidelity Taq, lane 9= positive control *egt2b* (size 1.55 kb) from *p423-ADH-egt2b*, lane 10= negative control.

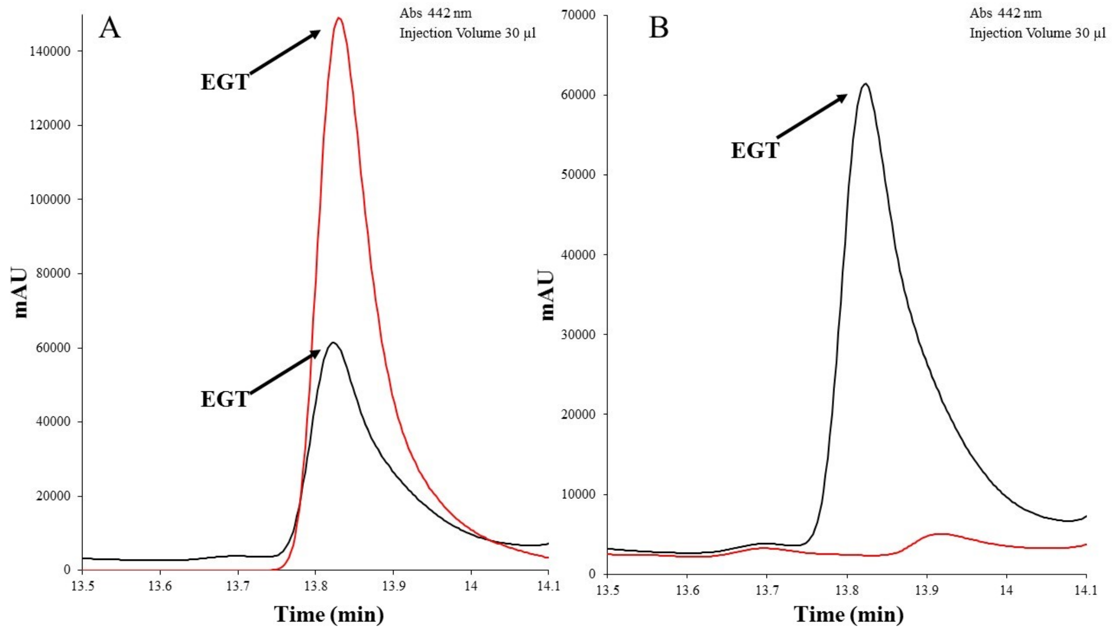

Supplementary Figure S3. D. Confirmation of EGT production in BY4741*^{egtAegt2a}*. (A) EGT in BY4741*^{egtAegt2a}* at a retention time of 13.84 min(black) compared to the EGT standard 13.85 min(red). (B) EGT in BY4741*^{egtAegt2a}* at a retention time of 13.84 min (black) compared to the BY4741*$^{p426\text{-}GPD\ p423\ ADH}$* no EGT is produced.

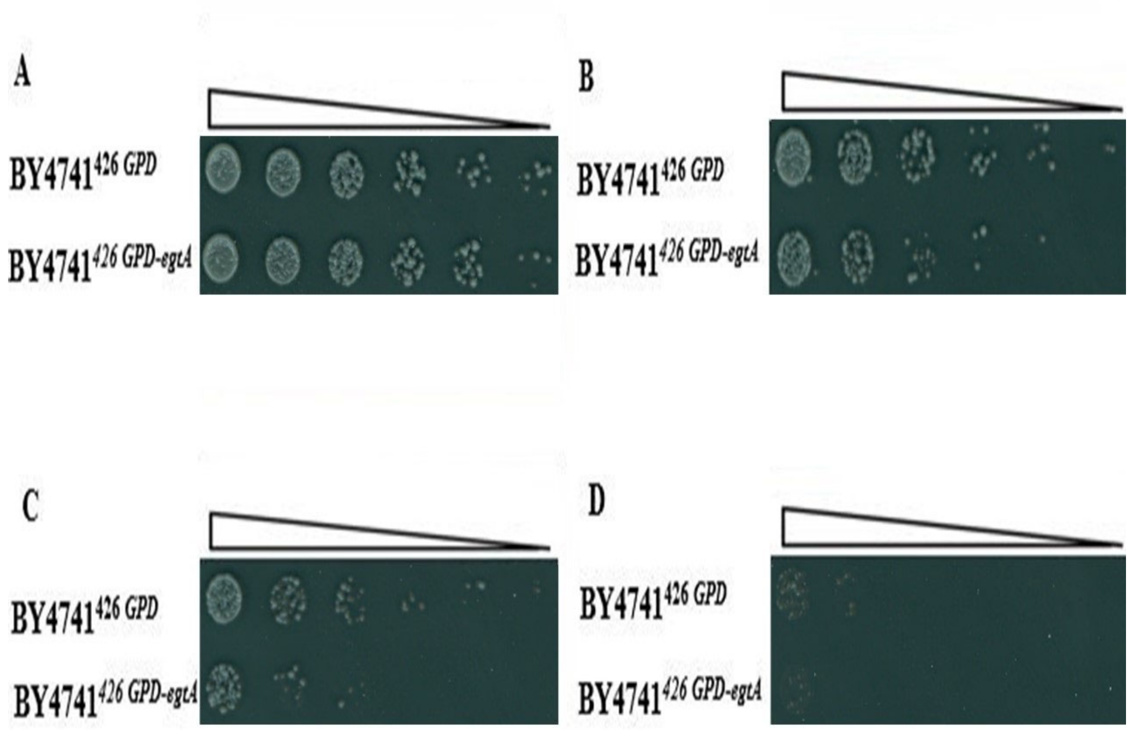

Supplementary Figure S4. Growth analysis of S. cerevisiae strains on synthetic complete media (SC) with hydrogen peroxide. Comparative growth analysis assay using strains BY4741$^{426\text{-}GPD}$ and BY4741$^{426\text{-}GPD\text{-}egtA}$. (A) Strains BY4741$^{426\text{-}GPD}$ and BY4741$^{426\text{-}GPD\text{-}egtA}$ grown on SC media. (B) Strains BY4741$^{426\text{-}GPD}$ and BY4741$^{426\text{-}GPD\text{-}egtA}$ grown on SC media with 1 mM hydrogen peroxide added. (C) Strains BY4741$^{426\text{-}GPD}$ and BY4741$^{426\text{-}GPD\text{-}egtA}$ grown on SC media with 1.5 mM hydrogen peroxide added. (D) Strains BY4741$^{426\text{-}GPD}$ and BY4741$^{426\text{-}GPD\text{-}egtA}$ grown on SC media with 2 mM hydrogen peroxide added.

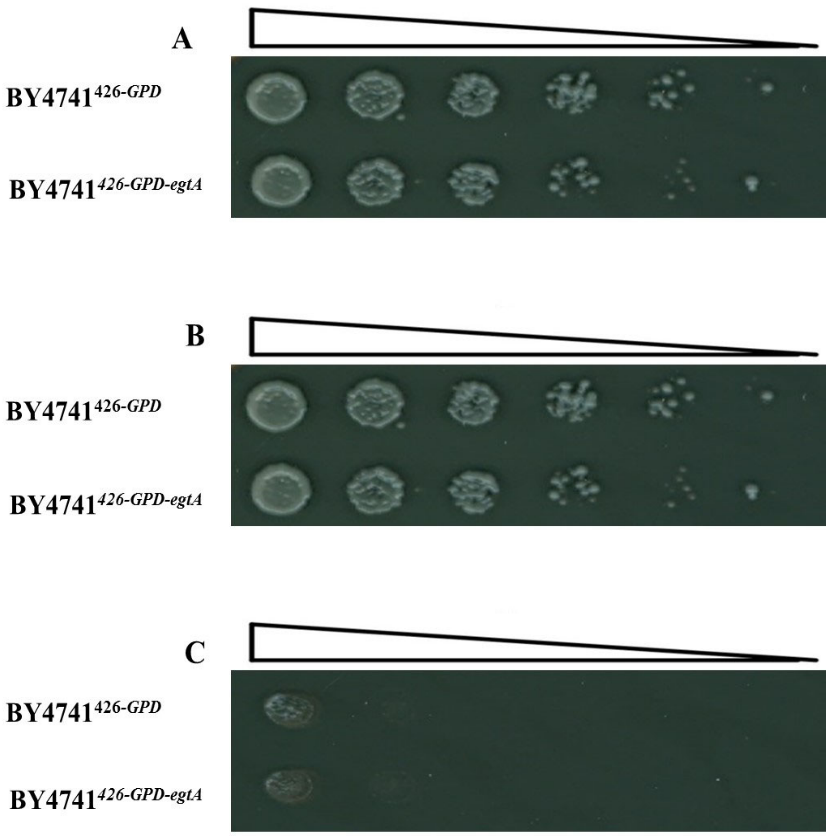

Supplementary Figure S5. Growth analysis of S. cerevisiae strains on synthetic complete media (SC) with ethanol. (A) Strains BY4741$^{426\text{-}GPD}$ and BY4741$^{426\text{-}GPD\text{-}egtA}$ grown on SC media. (B) Strains BY4741$^{426\text{-}GPD}$ and BY4741$^{426\text{-}GPD\text{-}egtA}$ grown on SC media with 4 % ethanol added. (C) Strains BY4741$^{426\text{-}GPD}$ and BY4741$^{426\text{-}GPD\text{-}egtA}$ grown on SC media with 8 % ethanol added.

Supplementary Table S2. Primer sequences used.

Primer name	Sequence (5′-3′)
EgtA F	AAATTTACTAGTATGTCCCCGTTGCCG
EgtA R	AATTCCCTCGAGCTACTGGCTGCGCACG
EgtA Internal F	AGAACCTACAGCGTCCCACTG
EgtA Internal R	CTTGACCTCCTCATAAGTGGG
Egt-2a F	AGATGCACTAGTATGTCCGCTCCCACA
Egt-2a R	AAGTCAGAATTCTCACAACTTTGGCTC
Egt-2b F	GGGCCCACTAGTATGTCTAGCGTTACGCCATCT
Egt-2b R	GGGCCCGAATTCCTAATGCTGACTCCATTGGAT
EgtA N-terminal His tag	AGAACTAGTATGCATCATCACCACCACCATATGTCCCCGTTGCCGTGTCC
Egt-2a N-terminal His tag	GGGCCCACTAGTATGCATCATCACCACCACCATATGTCCGCTCCCACA
Egt-2b N-terminal His tag	GGGCCCACTAGTATGCATCATCACCACCACCATATGTCTAGCGTTACGCCATC

Egt-2b C-terminal His tag	GAGGAATTCCTAATGGTGGTGGTGATGATGATGCTGACTCCATTGGAT
Pc210 F	CAACCAACCCTTTTACGGTCTC
Pc210 R	AATTAGTACGGGCGTGTGGTCT
ADH F	GTTTCCTCGTCATTGTTCTCG
GPD F	CGGTAGGTATTGATTGTAATTCTG
